# Long-Term Cardiovascular and Cerebrovascular Challenges Posed by COVID-19 in Patients With Familial Hypercholesterolemia

**DOI:** 10.3389/fphar.2022.890141

**Published:** 2022-05-11

**Authors:** Alpo Vuorio, Frederick Raal, Petra Ijäs, Markku Kaste, Petri T. Kovanen

**Affiliations:** ^1^ Mehiläinen, Airport Health Center, Vantaa, Finland; ^2^ Department of Forensic Medicine, University of Helsinki, Helsinki, Finland; ^3^ Faculty of Health Sciences, University of Witwatersrand, Johannesburg, South Africa; ^4^ University of Helsinki and Helsinki University Hospital, Helsinki, Finland; ^5^ Wihuri Research Institute, Helsinki, Finland

**Keywords:** long-covid, endothelial dysfunction, familial hypercholesterolemia, PCSK9 inhibitor, statin, stroke, CHD

## Introduction

Follow-up studies have revealed that during the severe acute respiratory syndrome (SARS) caused by coronavirus SARS-CoV epidemic between 2002 and 2003, a significant proportion of those infected had a deterioration in the general health and exercise capacity, especially during the 24 months post-infection (Ngai et al., 2010; Zhang et al., 2020a). Although the emphasis of the follow-up by Ngai and coworkers (2010) was on monitoring alterations in the lung function, a significant impairment in quality of life, as assessed by the SF-36 quality-of-life measures, was reported (Ngai et al., 2010). The long-term effects of SARS-CoV infection have been found to also affect lipid and glucose metabolism in recovered SARS patients (Wu et al., 2017). Thus, the impaired metabolic pathologies were observed 12 years after the infection, and they were considered to be long-term effects of the initial lung damages, and to be potentially also related to the one-month-long high-dose prednisolone treatment given during the acute phase of the disease. Long-term symptoms, usually lasting for about 24 months, have also been reported in survivors of the epidemic influenza A (H7N9) (Chen et al., 2017) and Dengue (Garcia et al., 2011). As could have been envisaged, numerous COVID-19 patients also suffer from long-term impairment of health after an acute SARS-CoV-2 infection, particularly if the symptoms of the disease have been severe and the patients had to be hospitalized (Nalbandian et al., 2021).

In an earlier commentary, we have discussed the acute phase of COVID-19 in patients with familial hypercholesterolemia (FH) (Vuorio et al., 2021a). We now wish to highlight concerns related to the long-term effects of SARS-CoV-2 infections and the increases in the risk for complications and potentially a poor outcome in this group of patients.

## Familial Hypercholesterolemia and COVID-19

FH is the most common monogenic inherited metabolic disease worldwide, and it affects an estimated one in every 330 individuals (Beheshti et al., 2020). Accordingly, we can estimate that among the currently reported over 500 million total cases of COVID-19 worldwide, more than one million are likely FH patients. FH patients have a markedly elevated serum low-density lipoprotein cholesterol (LDL-C) already *in utero* which is often accompanied by an elevated level of serum lipoprotein(a) [Lp(a)], and if left untreated, a lifelong dysfunction of the arterial endothelium ensues in these patients (Sorensen et al., 1994; Vuorio et al., 2020; Nurmohamed et al., 2022). In FH patients with COVID-19, the pre-existing endothelial dysfunction is likely to increase the risk of macrovascular and microvascular thrombosis caused by a direct viral attack of the endothelial cells (endothelitis) and by the cytokine storm typically seen during severe COVID-19 illness (Vuorio et al., 2021b). In FH patients, such compounded endothelial injuries with ensuing thrombosis/thromboembolism pose a danger to cardiovascular health, particularly when they affect the coronary vessels supplying the heart and/or the extracranial and intracranial vessels supplying the brain (Quick et al., 2021). Thus, severe COVID-19 illness has been found to increase the risk of myocardial infarction in FH patients whether they have or have not been diagnosed with atherosclerotic cardiovascular disease (ASCVD) (Myers et al., 2021). Additionally, the elevated risk of ischemic stroke associated with COVID-19 potentially also applies to FH patients with COVID-19 (Vuorio et al., 2021c).

## Long COVID or Post-COVID Syndrome

Long COVID or the post-COVID-19 syndrome has been defined as a condition characterized by persistent symptoms lasting more than 2 months after the onset of SARS-CoV-2 infection (Soriano et al., 2021). The severity of the post-COVID syndrome is highly variable, and research suggests that even persons with mild COVID-19, i.e., nearly asymptomatic SARS-CoV-2 infection, may experience long-term symptoms ranging from neuropsychiatric symptoms, such as chronic fatigue, to arterial, venous, and microvascular thrombotic complications in various organs (Di Toro et al., 2021; Nalbandian et al., 2021). In fact, because the long COVID symptoms are frequent among COVID-19 patients (about 30% or more) who have been hospitalized, the American Heart Foundation has initiated an extensive program in order to determine if those individuals who have ASCVD or have survived stroke are more prone to develop long COVID (https://newsroom.heart.org/news/10-million-invested-to-study-long-term-impact-of-covid-19-on-heart-and-brain-health).

## Potential Threats to Cardiac Function After SARS-CoV-2 Infection

Although research into the etiological factors of long COVID needs further study, some causal mechanisms potentially relevant also for FH patients with long COVID have already been identified, as discussed below. However, it is important to remember that the data refer to a very wide variety of patients with SARS-CoV-2 infection, and, accordingly, the clinicians need to be careful when trying to generalize the results so far available. Moreover, no long-term cardiovascular or cerebrovascular clinical data on FH patients after COVID-19 have yet been published; so, we have to utilize indirect measures when assessing the significance of the results already obtained in non-FH patients.

One potentially interesting cause of dyspnea due to COVID-19 was found in patients without a known pre-existing cardiopulmonary disease (mean age 51 ± 11 years), and who underwent a 1-year intensive clinical follow-up (Luchian et al., 2022). All of the patients had persistent dyspnea, and in more than a third, the echocardiographic evaluation revealed significant changes in cardiac global constructive work and global work index, suggesting that these patients had decreased myocardial performance and a subclinical cardiac dysfunction. Earlier studies have suggested that dyspnea in the recovered patients who suffered SARS-CoV-2 infection could be related to myocarditis or ischemic injury of the heart (Kotecha et al., 2021; Özer et al., 2021). Regarding FH patients, COVID-19-associated impairment in myocardial performance is highly relevant, particularly among those patients with pre-existing ASCVD, a condition which is often present in untreated FH patients already in early adulthood, even if left undiagnosed (Representatives of the Global Familial Hypercholesterolemia Community, 2020). It is possible that decreased myocardial performance continuing after COVID-19 potentially affects a much wider range of FH patients, including those without clinically evident ASCVD. A very recent study among 46 male FH patients without known ASCVD (and without COVID-19) demonstrated a mildly reduced global longitudinal left ventricular strain when compared with controls (Vartela et al., 2021). This finding suggests that, particularly in FH males, the presence of subclinical vasculopathy adversely affecting the cardiac function might predispose the patients to even greater deleterious post-COVID-19 sequelae.

## Convalescent Period of COVID-19 and the Potential Risk of Ischemic Stroke and Intracranial Hemorrhage

In an early study on neurological and psychiatric sequelae of COVID-19, the risk of cerebrovascular events, notably acute ischemic stroke and intracranial hemorrhage, was found to be significantly increased within the first 6 months after COVID-19, particularly in patients who had suffered from severe acute COVID-19 and associated encephalopathy (Taquet et al., 2021). In a study of South Asian males aged 50 years or younger, the estimated annual incidence rate of acute ischemic stroke was significantly higher (82.6 cases per 100,000 persons) in those with COVID-19 infection when compared with historical data (38.2 cases per 100,000 persons) (Tu et al., 2021). Importantly, in this study, acute ischemic stroke was reported to occur during the convalescent phase after an asymptomatic COVID-19 infection, the median time from a positive serological test result to stroke being 55 days (range 0–130 days).

Bikdeli and coworkers (2020) have reported acute ischemic stroke as a secondary wave of complications of COVID-19 and postulated that the prothrombotic state associated with acute COVID-19 may persist long-term (Bikdeli et al., 2020). A recent study utilizing healthcare databases from the US Department of Veterans Affairs indicated that the period of increased risk and burden for cerebrovascular disorders (stroke and transient ischemic attack) may persist at least for 1 year after the infection (Xie et al., 2022). Thus, the authors reported that persons who survived the first 30 days of COVID-19 exhibited an increased risk of stroke and transient ischemic attack for 12 months after the acute infection. In this register study, the exact mechanisms underlying cerebrovascular events remained undetermined, but the high incidence of high-risk cardioembolic conditions (atrial fibrillation, heart failure, acute coronary syndrome, myocarditis) suggests that strokes may be secondary to cardiac disease, while other COVID-19-related mechanisms (hypercoagulopathy, endotheliitis) likely contribute to the final events.

Regarding hypercoagulability, elevated levels of plasma factor VII and plasminogen activator inhibitor-1 have been shown to persist after SARS-CoV-2 infection (von Meijenfeldt et al., 2021). There is also a concern about coagulopathy and the appearance of antiphospholipid antibodies, which can arise transiently in patients with various infections including COVID-19 (Zhang et al., 2020b). Such antibodies, particularly the anticardiolipin antibodies, may have an acute ischemic stroke risk-impacting effect jointly with other well-recognized risk factors for stroke, such as hypertension, hyperlipidemia, and obesity (Rothstein et al., 2020). What could such scenarios potentially mean in the context of FH? An early study found that untreated FH patients, i.e., those not receiving lipid-lowering therapy, have a markedly increased risk for acute ischemic stroke (Kaste and Koivisto, 1988) More recently, i.e., during the statin era, such increased risk appears to have largely disappeared, most likely reflecting an effective treatment of the hypercholesterolemia (Huxley et al., 2003; Soljanlahti et al., 2005; Hovland et al., 2018; Beheshti et al., 2018). Unfortunately, however, the great majority of FH patients have not been diagnosed, and, accordingly, they remain untreated or, even if correctly diagnosed, remain undertreated (Representatives of the Global Familial Hypercholesterolemia Community, 2020). Therefore, the concern regarding the risk of an acute ischemic stroke in most FH patients with COVID-19 continues.

## A Persistent Hypercoagulable State After SARS-CoV-2 Infection

A recently reported South African study was searching for a common explanator of the large variety of long COVID symptoms (Pretorius et al., 2021). The study included patients who had suffered from long COVID for at least 2 months, and as control groups healthy persons and patients with type 2 diabetes mellitus without known previous SARS-CoV-2 infection. Although the study was small in size, it revealed that the plasma derived from the long COVID patients contained large anomalous deposits of microclots. After trypsinization of the plasma, increased concentrations of several pro-inflammatory molecules such as alpha (2)-antiplasmin, various fibrinogen chains, and serum amyloid A (SAA) were detected in the samples derived from the long COVID patients, but not in those from the healthy or diabetic control subjects. The authors concluded that the clotting proteins were dysfunctional and that an imbalance between the supply and demand of lytic enzymes existed. Three mechanisms were postulated as an explanation of the clotting pathology in the long COVID-19 patients: first, the increased levels of pro-inflammatory molecules in the plasma caused a hypercoagulable state; second, platelets were hyperactivated and led to microclot formation in the circulation, and third, an aberrant fibrinolytic system prevailed. Based on their findings, the authors recommended considering prolonged anticoagulation for COVID-19 patients after discharge from the hospital. Regarding the FH patients, an elevated serum Lp(a) is also a matter of major concern because the unique apolipoprotein(a) component present in the Lp(a) particles prevents fibrinolysis and so tends to promote thrombus growth wherever the thrombus is forming in the vasculature (Vuorio et al., 2020).

## Concluding Remarks

When compared with non-FH patients who have suffered an acute SARS-CoV-2 infection, in FH patients a hypercoagulable state may persist for even longer periods after the infection. This assumption is relevant because the endothelial cells have been exposed to a lifelong high LDL-C concentration, and often also to an elevated Lp(a) level, which jointly cause endothelial dysfunction even in childhood (Vuorio et al., 2021b). This can be particularly harmful in the FH patients whose LDL-C-lowering therapy is lacking or sub-optimal, and among those FH patients who also have a highly elevated serum Lp(a) level. Thrombus formation in an arterial, venous, or microvascular vascular segment, is likely to occur with greater frequency among FH patients not only because of a pre-existing endothelial dysfunction but also as a result of the acute direct viral endothelial damage and the hypercoagulability state during the post-COVID period.

The COVID-19 pandemic will increase health inequalities, and particularly in low-income countries, there is a need to increase vaccination coverage in the most vulnerable patient groups (Vuorio et al., 2022). Significant health inequalities between high-to-low-income countries are demonstrated by the fact that by April 2022, only about 15% of people in low-income countries had received at least one dose of a COVID-19 vaccine (https://www.ourworldindata.org/covid-vaccinations). The COVID-19 vaccination program is important not only to prevent acute SARS-CoV-2 infections but also to potentially decrease long-term COVID-19 complications.

Although reliable epidemiological data are not yet available, it is obvious that the entire FH population, i.e., about 30 million FH patients worldwide, are at an increased risk of serious vascular complications which can occur after COVID-19. Consequently, when a clinician encounters an FH patient with symptoms that match with those typical of the post-COVID syndrome, it is of importance that the clinician ascertains that lipid-lowering pharmacotherapy is being taken regularly, is adequate, and follows the current guidelines. In this context, it is relevant to remember that statins also act as mild antithrombotic medications (Vuorio et al., 2021d). When appropriate, PCSK9 inhibition may be included in the lipid-lowering therapy even among younger FH patients with COVID-19 and thereafter, as, unlike statins, the PCSK9 inhibitors can also reduce the level of serum Lp(a) (Vuorio et al., 2020).

## References

[B1] Beheshti S. Madsen C. M. Varbo A. Benn M. Nordestgaard B. G. (2018). Relationship of Familial Hypercholesterolemia and High Low-Density Lipoprotein Cholesterol to Ischemic Stroke: Copenhagen General Population Study. Circulation 138 (6), 578–589. 10.1161/CIRCULATIONAHA.118.033470 PubMed Abstract | 10.1161/CIRCULATIONAHA.118.033470 | Google Scholar 29593013

[B2] Beheshti S. O. Madsen C. M. Varbo A. Nordestgaard B. G. (2020). Worldwide Prevalence of Familial Hypercholesterolemia: Meta-Analyses of 11 Million Subjects. J. Am. Coll. Cardiol. 75 (20), 2553–2566. 10.1016/j.jacc.2020.03.057 PubMed Abstract | 10.1016/j.jacc.2020.03.057 | Google Scholar 32439005

[B3] Bikdeli B. Madhavan M. V. Jimenez D. Chuich T. Dreyfus I. Driggin E. (2020). COVID-19 and Thrombotic or Thromboembolic Disease: Implications for Prevention, Antithrombotic Therapy, and Follow-Up: JACC State-Of-The-Art Review. J. Am. Coll. Cardiol. 75 (23), 2950–2973. 10.1016/j.jacc.2020.04.031 PubMed Abstract | 10.1016/j.jacc.2020.04.031 | Google Scholar 32311448 PMC7164881

[B4] Chen J. Wu J. Hao S. Yang M. Lu X. Chen X. (2017). Long Term Outcomes in Survivors of Epidemic Influenza A (H7N9) Virus Infection. Sci. Rep. 7 (1), 17275. 10.1038/s41598-017-17497-6 PubMed Abstract | 10.1038/s41598-017-17497-6 | Google Scholar 29222500 PMC5722861

[B5] Di Toro A. Bozzani A. Tavazzi G. Urtis M. Giuliani L. Pizzoccheri R. (2021). Long COVID: Long-Term Effects? Eur. Heart J. Suppl. 23 (Suppl. E), E1–E5. 10.1093/eurheartj/suab080 PubMed Abstract | 10.1093/eurheartj/suab080 | Google Scholar 34650349 PMC8503490

[B6] García G. González N. Pérez A. B. Sierra B. Aguirre E. Rizo D. (2011). Long-term Persistence of Clinical Symptoms in Dengue-Infected Persons and its Association with Immunological Disorders. Int. J. Infect. Dis. 15 (1), e38–e43. 10.1016/j.ijid.2010.09.008 PubMed Abstract | 10.1016/j.ijid.2010.09.008 | Google Scholar 21112804

[B7] Kaste M. Koivisto P. (1988). Risk of Brain Infarction in Familial Hypercholesterolemia. Stroke 19 (9), 1097–1100. 10.1161/01.str.19.9.1097 PubMed Abstract | 10.1161/01.str.19.9.1097 | Google Scholar 3413806

[B8] Kotecha T. Knight D. S. Razvi Y. Kumar K. Vimalesvaran K. Thornton G. (2021). Patterns of Myocardial Injury in Recovered Troponin-Positive COVID-19 Patients Assessed by Cardiovascular Magnetic Resonance. Eur. Heart J. 42 (19), 1866–1878. 10.1093/eurheartj/ehab075 PubMed Abstract | 10.1093/eurheartj/ehab075 | Google Scholar 33596594 PMC7928984

[B9] Luchian M.-L. Motoc A. Lochy S. Magne J. Belsack D. De Mey J. (2022). Subclinical Myocardial Dysfunction in Patients with Persistent Dyspnea One Year after COVID-19. Diagnostics 12 (1), 57. 10.3390/diagnostics12010057 10.3390/diagnostics12010057 | Google Scholar PMC8775030 35054224

[B10] Myers K. D. Wilemon K. McGowan M. P. Howard W. Staszak D. Rader D. J. (2021). COVID-19 Associated Risks of Myocardial Infarction in Persons with Familial Hypercholesterolemia with or without ASCVD. Am. J. Prev. Cardiol. 7, 100197. 10.1016/j.ajpc.2021.100197 PubMed Abstract | 10.1016/j.ajpc.2021.100197 | Google Scholar 34611637 PMC8387291

[B11] Nalbandian A. Sehgal K. Gupta A. Madhavan M. V. McGroder C. Stevens J. S. (2021). Post-acute COVID-19 Syndrome. Nat. Med. 27 (4), 601–615. 10.1038/s41591-021-01283-z PubMed Abstract | 10.1038/s41591-021-01283-z | Google Scholar 33753937 PMC8893149

[B12] Ngai J. C. Ko F. W. Ng S. S. To K. W. Tong M. Hui D. S. (2010). The Long-Term Impact of Severe Acute Respiratory Syndrome on Pulmonary Function, Exercise Capacity and Health Status. Respirology 15 (3), 543–550. 10.1111/j.1440-1843.2010.01720.x PubMed Abstract | 10.1111/j.1440-1843.2010.01720.x | Google Scholar 20337995 PMC7192220

[B13] Nurmohamed N. S. Collard D. Reeskamp L. F. Kaiser Y. Kroon J. Tromp T. R. (2022). Lipoprotein(a), Venous Thromboembolism and COVID-19: A Pilot Study. Atherosclerosis 341, 43–49. 10.1016/j.atherosclerosis.2021.12.008 PubMed Abstract | 10.1016/j.atherosclerosis.2021.12.008 | Google Scholar 34995986 PMC8690577

[B14] Özer S. Candan L. Özyıldız A. G. Turan O. E. (2021). Evaluation of Left Ventricular Global Functions with Speckle Tracking Echocardiography in Patients Recovered from COVID-19. Int. J. Cardiovasc. Imaging 37 (7), 2227–2233. 10.1007/s10554-021-02211-5 PubMed Abstract | 10.1007/s10554-021-02211-5 | Google Scholar 33725265 PMC7961169

[B15] Pretorius E. Vlok M. Venter C. Bezuidenhout J. A. Laubscher G. J. Steenkamp J. (2021). Persistent Clotting Protein Pathology in Long COVID/Post-Acute Sequelae of COVID-19 (PASC) Is Accompanied by Increased Levels of Antiplasmin. Cardiovasc Diabetol. 20 (1), 172. 10.1186/s12933-021-01359-7 PubMed Abstract | 10.1186/s12933-021-01359-7 | Google Scholar 34425843 PMC8381139

[B16] Quick S. Moss J. Rajani R. M. Williams A. (2021). A Vessel for Change: Endothelial Dysfunction in Cerebral Small Vessel Disease. Trends. Neurosci. 44 (4), 289–305. 10.1016/j.tins.2020.11.003 PubMed Abstract | 10.1016/j.tins.2020.11.003 | Google Scholar 33308877

[B17] Rothstein A. Oldridge O. Schwennesen H. Do D. Cucchiara B. L. (2020). Acute Cerebrovascular Events in Hospitalized COVID-19 Patients. Stroke 51 (9), e219–e222. 10.1161/STROKEAHA.120.030995 PubMed Abstract | 10.1161/STROKEAHA.120.030995 | Google Scholar 32684145 PMC7386677

[B18] Soljanlahti S. Autti T. Lauerma K. Raininko R. Keto P. Turtola H. (2005). Familial Hypercholesterolemia Patients Treated with Statins at No Increased Risk for Intracranial Vascular Lesions Despite Increased Cholesterol Burden and Extracranial Atherosclerosis. Stroke 36 (7), 1572–1574. 10.1161/01.STR.0000169920.64180.fa PubMed Abstract | 10.1161/01.STR.0000169920.64180.fa | Google Scholar 15933262

[B19] Sorensen K. E. Celermajer D. S. Georgakopoulos D. Hatcher G. Betteridge D. J. Deanfield J. E. (1994). Impairment of Endothelium-dependent Dilation Is an Early Event in Children with Familial Hypercholesterolemia and Is Related to the Lipoprotein(a) Level. J. Clin. Invest 93 (1), 50–55. 10.1172/JCI116983 PubMed Abstract | 10.1172/JCI116983 | Google Scholar 8282821 PMC293724

[B20] Soriano J. B. Murthy S. Marshall J. C. Relan P. Diaz J. V. WHO Clinical Case Definition Working Group on Post-COVID-19 Condition (2021). A Clinical Case Definition of Post-COVID-19 Condition by a Delphi Consensus. Lancet Infect. Dis. 22 (4), 00703–00709. 10.1016/S1473-3099(21)00703-9: 10.1016/S1473-3099(21)00703-9 | Google Scholar PMC8691845 34951953

[B21] Taquet M. Geddes J. R. Husain M. Luciano S. Harrison P. J. (2021). 6-month Neurological and Psychiatric Outcomes in 236 379 Survivors of COVID-19: a Retrospective Cohort Study Using Electronic Health Records. Lancet Psychiatry 8 (5), 416–427. 10.1016/S2215-0366(21)00084-5 PubMed Abstract | 10.1016/S2215-0366(21)00084-5 | Google Scholar 33836148 PMC8023694

[B22] Tu T. M. Seet C. Y. H. Koh J. S. Tham C. H. Chiew H. J. De Leon J. A. (2021). Acute Ischemic Stroke during the Convalescent Phase of Asymptomatic COVID-2019 Infection in Men. JAMA Netw. Open 4 (4), e217498. 10.1001/jamanetworkopen.2021.7498 PubMed Abstract | 10.1001/jamanetworkopen.2021.7498 | Google Scholar 33885771 PMC8063067

[B23] Vartela V. Armenis I. Leivadarou D. Toutouzas K. Makrilakis K. Athanassopoulos G. D. (2021). Reduced Global Longitudinal Strain at Rest and Inadequate Blood Pressure Response during Exercise Treadmill Testing in Male Heterozygous Familial Hypercholesterolemia Patients. Int. J. Cardiol. Hypertens. 9, 100083. 10.1016/j.ijchy.2021.100083 PubMed Abstract | 10.1016/j.ijchy.2021.100083 | Google Scholar 34095810 PMC8167294

[B24] von Meijenfeldt F. A. Havervall S. Adelmeijer J. Lundström A. Magnusson M. Mackman N. (2021). Sustained Prothrombotic Changes in COVID-19 Patients 4 Months after Hospital Discharge. Blood Adv. 5 (3), 756–759. 10.1182/bloodadvances.2020003968 PubMed Abstract | 10.1182/bloodadvances.2020003968 | Google Scholar 33560386 PMC7857699

[B25] Vuorio A. Kaste M. Kovanen P. T. (2021c). Familial Hypercholesterolemia and Statins in the COVID-19 Era: Mitigating the Risk of Ischemic Stroke. eNeurologicalSci 23, 100344. 10.1016/j.ensci.2021.100344 PubMed Abstract | 10.1016/j.ensci.2021.100344 | Google Scholar 33937536 PMC8078044

[B26] Vuorio A. Kovanen P. T. Santos R. D. Raal F. (2022). Prevention of Cardiovascular Burden in COVID-19 Patients Suffering from Familial Hypercholesterolemia: A Global Challenge. Cardiol. Ther. 11, 1–7. 10.1007/s40119-021-00245-3 PubMed Abstract | 10.1007/s40119-021-00245-3 | Google Scholar 34787816 PMC8596860

[B27] Vuorio A. Lassila R. Kovanen P. T. (2021d). Hypercholesterolemia and COVID-19: Statins for Lowering the Risk of Venous Thromboembolism. Front. Cardiovasc. Med. 8, 711923. 10.3389/fcvm.2021.711923 PubMed Abstract | 10.3389/fcvm.2021.711923 | Google Scholar 34722654 PMC8548371

[B28] Vuorio A. Raal F. Kaste M. Kovanen P. T. (2021b). Familial Hypercholesterolaemia and COVID-19: A Two-Hit Scenario for Endothelial Dysfunction Amenable to Treatment. Atherosclerosis 320, 53–60. 10.1016/j.atherosclerosis.2021.01.021 10.1016/j.atherosclerosis.2021.01.021 | Google Scholar 33540179 PMC7830285

[B29] Vuorio A. Strandberg T. E. Raal F. Santos R. D. Kovanen P. T. (2021a). Familial Hypercholesterolemia and COVID-19: A Menacing but Treatable Vasculopathic Condition. Atheroscler. Plus 43, 3–6. 10.1016/j.athplu.2021.08.001 PubMed Abstract | 10.1016/j.athplu.2021.08.001 | Google Scholar 34622243 PMC8349422

[B30] Vuorio A. Watts G. F. Schneider W. J. Tsimikas S. Kovanen P. T. (2020). Familial Hypercholesterolemia and Elevated Lipoprotein(a): Double Heritable Risk and New Therapeutic Opportunities. J. Intern Med. 287 (1), 2–18. 10.1111/joim.12981 PubMed Abstract | 10.1111/joim.12981 | Google Scholar 31858669

[B31] Representatives of the Global Familial Hypercholesterolemia Community Wilemon K. A. Wilemon K. A. Patel J. Aguilar-Salinas C. Ahmed C. D. Alkhnifsawi M. (2020). Reducing the Clinical and Public Health Burden of Familial Hypercholesterolemia: A Global Call to Action. JAMA Cardiol. 5 (2), 217–229. 10.1001/jamacardio.2019.5173 PubMed Abstract | 10.1001/jamacardio.2019.5173 | Google Scholar 31895433

[B32] Wu Q. Zhou L. Sun X. Yan Z. Hu C. Wu J. (2017). Altered Lipid Metabolism in Recovered SARS Patients Twelve Years after Infection. Sci. Rep. 7 (1), 9110. 10.1038/s41598-017-09536-z PubMed Abstract | 10.1038/s41598-017-09536-z | Google Scholar 28831119 PMC5567209

[B33] Xie Y. Xu E. Bowe B. Al-Aly Z. (2022). Long-term Cardiovascular Outcomes of COVID-19. Nat. Med. 28, 583–590. 10.1038/s41591-022-01689-3 PubMed Abstract | 10.1038/s41591-022-01689-3 | Google Scholar 35132265 PMC8938267

[B34] Zhang P. Li J. Liu H. Han N. Ju J. Kou Y. (2020a). Long-term Bone and Lung Consequences Associated with Hospital-Acquired Severe Acute Respiratory Syndrome: a 15-year Follow-Up from a Prospective Cohort Study. Bone Res. 8, 8. 10.1038/s41413-020-0084-5 PubMed Abstract | 10.1038/s41413-020-0084-5 | Google Scholar 32128276 PMC7018717

[B35] Zhang Y. Xiao M. Zhang S. Xia P. Cao W. Jiang W. (2020b). Coagulopathy and Antiphospholipid Antibodies in Patients with Covid-19. N. Engl. J. Med. 382 (17), e38. 10.1056/NEJMc2007575 PubMed Abstract | 10.1056/NEJMc2007575 | Google Scholar 32268022 PMC7161262

